# Impact of biogas slurry on soil health, organic carbon, soil acidification, and soybean yield in Northeast China’s black soils

**DOI:** 10.3389/fmicb.2026.1805527

**Published:** 2026-04-29

**Authors:** N’Dri Yves Bohoussou, Olouwatogni Michaël Ayénikafo, Guoxiang Zheng, Lei Wang, Wenbo Wu, Fengtao Ju, Yao Dinard Kouadio, Ahmad Latif Virk, Yash Pal Dang, Ming Wang

**Affiliations:** 1 College of Engineering, Northeast Agriculture University, Harbin, China; 2 Key Laboratory of Agricultural Renewable Resources Utilization Technology and Equipment in Cold Areas of Heilongjiang Province, Harbin, China; 3 Agricultural Economics and Management, Northeast Agricultural University, Harbin, China; 4 Key Laboratory of Swine Facilities Engineering, Ministry of Agriculture and Rural Affairs, Harbin, China; 5 Laboratory Economics and Rural Development, Institute of Agropastoral Management, Peleforo GON COULIBALY University of Korhogo, Korhogo, Côte d’Ivoire; 6 College of Ecology and Environment, Chengdu University of Technology, Chengdu, China; 7 School of Agriculture and Food Sustainability, The University of Queensland, St Lucia, QLD, Australia

**Keywords:** biogas slurry, black soil, carbon storage, crop productivity, microbial community, soil pH

## Abstract

Black soils in Northeast China are among the most fertile globally, but have experienced significant degradation due to long-term intensive use of chemical fertilizers. Although organic amendments such as biogas slurry are considered promising alternatives, their effects on black soils in this region remain underexplored. In this study, a controlled pot experiment was conducted to evaluate the effect of biogas slurry on soil health, soil organic carbon (SOC), total nitrogen (TN), soil acidification, and soybean yield. In our study, biogas slurry was derived from cow dung. Four treatments were applied: no fertilizer (CK), chemical fertilizer (N), biogas slurry (BS), and mixed fertilizer (BS + N). Our study revealed that fertilizer application showed a significant (P < 0.05) impact on soil organic carbon (SOC) and total phosphorus (TP). The highest SOC (8.97 g/kg) and TN (1.30 g/kg) were observed under BS treatment. The soil pH was significantly (P < 0.05) higher under BS (6.69) and BS+N (6.64) than under N (6.40), indicating a buffering effect. Microbial biomass carbon (MBC) and nitrogen (MBN) were significantly enhanced under BS and BS+N, while N alone reduced MBN (*P* < 0.05). BS significantly increased soil Cu (38.66mg/kg) and Cd (0.13mg/kg) concentrations, whereas Zn and Pb remained unchanged. Soybean yield was significantly affected by fertilization, with the highest yield observed under the BS+N treatment (1.65 Mg/ha), followed by chemical fertilizer (1.35 Mg/ha). Overall, biogas slurry improved soil fertility indicators and mitigated soil acidification under the experimental conditions, while mixed fertilizer provided the greatest yield benefits. However, these findings are based on short-term pot conditions, and long-term field studies are still required to confirm the potential environmental risks of biogas slurry application.

## 1 Introduction

The Northeast region of China is one of the country’s primary agricultural strongholds, characterized by extensive areas of fertile black soil, covering approximately 1,030,000 km^2^ (Liu et al., 2012; Xu et al., 2010). These black soils are characterized by their high organic matter content, particularly soil organic carbon (SOC), and a well-structured composition that promotes water and nutrient retention (Zhang et al., 2023). Their neutral to slightly acidic pH, combined with high levels of nitrogen, phosphorus, and potassium, makes them particularly suitable for growing cereals such as corn and soybeans (Zhou et al., 2021). As such, this region makes a significant contribution to national grain production and plays a vital role in ensuring China’s food security (Gao et al., 2022). However, decades of intensive farming and overreliance on chemical fertilizers have led to widespread degradation of these soils. Unsustainable agricultural practices have resulted in declining SOC levels, soil acidification, and reduced crop productivity (Lin et al., 2023). The continued degradation of black soils poses a significant challenge to sustainable agriculture, threatening efforts to ensure stable and long-term food production (Wang H. et al., 2022). In this context, optimizing fertilization practices is essential for maintaining soil fertility, regulating nutrient cycling, and enhancing crop yields, particularly in soybean-based production systems.

While chemical fertilizers have contributed substantially to yield gains over recent decades, their overuse has had adverse ecological effects. These include increased soil acidification, reductions in SOC and TN, and disruption to microbial activities and overall soil health (Ding et al., 2022). In contrast, integrated nutrient management practices, such as mixed fertilization, combining organic and inorganic fertilizers, have been shown to balance crop nutrient needs while improving soil quality (Niu et al., 2022). This approach mitigates the negative effects of excessive chemical input and enhances soybean yields, and supports sustainable agricultural systems. Mixed fertilization improves SOC and TN, enhances soil structure, promotes microbial activity, and helps maintain a stable soil pH, thereby reducing soil acidification (Bohoussou et al., 2023; Zhang et al., 2024).

Fertilization not only influences soil fertility but can also alter the dynamics of heavy metal accumulation in soil. Both chemical and organic fertilizers introduce trace amounts of heavy metals such as copper (Cu), zinc (Zn), lead (Pb), and cadmium (Cd), either through impurities in mineral fertilizers or biogas slurries (Sun et al., 2023). In the context of Northeast China’s black soils, characterized by high organic matter content, strong cation exchange capacity, and significant buffering ability, these heavy metals may be retained in the topsoil or mobilized depending on soil pH and organic complexation (Li Q. et al., 2022). While moderate levels of Cu and Zn are essential for plant growth, excessive accumulation can lead to phytotoxicity and long-term environmental risks (Alengebawy et al., 2021; Guéablé et al., 2025). Cd and Pb, even at low concentrations, are particularly concerning due to their mobility, persistence, and potential for food chain transfer (Yin et al., 2024). Thus, understanding how different fertilizer strategies, especially biogas slurry, affect heavy metal dynamics in black soils is essential for balancing soil health and environmental safety in sustainable agricultural systems. This is particularly relevant for organic amendments, which can simultaneously improve soil fertility and introduce trace elements into the soil system.

In this context, organic amendments have received increasing attention as sustainable alternatives to chemical fertilizers due to their potential to improve soil structure, nutrient cycling, and microbial activity. Soybean crops, in particular, require well-balanced nutrient input for optimal growth, and several studies revealed that mixed fertilizers enhance soil fertility and yield performance more effectively than mineral fertilizers alone (Qi et al., 2024; Xie et al., 2023). Among these organic amendments, biogas slurry, a byproduct of anaerobic digestion of organic residues, offers a promising organic amendment for sustainable soil management (Jain et al., 2015). Biogas slurry contains high levels of readily plant-available nutrients, including inorganic nitrogen, phosphorus, potassium, and dissolved organic carbon. Moreover, it contains an abundant and active microbial community, which can reinforce soil health (Wang et al., 2012). The application of biogas slurry has been reported to increase SOC, stimulate microbial biomass, and enhance nutrient mineralization processes (Niyungeko et al., 2020). Additionally, it counteracts soil acidification by modulating soil pH, thereby creating a more favorable environment for nutrient uptake (Zheng et al., 2017). When applied to soil, biogas slurry contributes to better soil aggregation, increased water storage capacity, and intensified microbial activity, promoting a conducive environment for plant growth. In Northeast China, biogas slurry has shown great potential in restoring soil fertility, enhancing soil resilience, and increasing crop yields, particularly for soybeans (Wang et al., 2023). Prior studies also indicate that biogas slurry can enhance nitrogen use efficiency, reduce nutrient leaching losses, and boost crop yield by improving both nutrient availability and microbial activity (Abubaker et al., 2013; Zou et al., 2025). The use of biogas slurry aligns with the principles of circular agriculture by recycling organic waste materials into productive inputs, reducing reliance on synthetic fertilizer (Yadav et al., 2023). While biogas slurry has been shown to increase SOC, TN, and microbial activities, some suggest that it may not always achieve the same level of crop yield enhancement as other fertilization strategies (Sieling et al., 2013; Wang et al., 2023).

Despite growing recognition of the agronomic and environmental benefits of biogas slurry, there is limited scientific evidence comparing its performance to chemical and mixed fertilizers, specifically in the black soil of Northeast China. This region, characterized by highly fertile Mollisols (black soils), is facing increasing challenges due to intensive farming practices and overuse of chemical fertilizers, which have led to soil degradation, acidification, and nutrient imbalance. Such as, the application of organic amendments, particularly biogas slurry, offers a potentially sustainable solution to counteract these negative impacts. This study addresses the effects of biogas slurry on soil fertility, microbial dynamics, and trace metal accumulation in these black soils. This study comprehensively evaluated the biogas slurry’s impact on microbial community shifts, heavy metal dynamics, and nutrient cycling. While biogas slurry has been studied in various soil types (Feng et al., 2024; Yin et al., 2025), its effects in Northeast China’s black soils remain underexplored.

Thus, this study aims to comparatively evaluate the effects of biogas slurry, mineral fertilizers, and their combined application on the fertility of black soils and soybean productivity. We hypothesize that biogas slurry alone or combined with chemical fertilizer could improve soil quality by increasing SOC, stabilizing pH, and promoting microbial activity, thereby improving soybean yields. The main objectives of this study were to; (i) determine the effects of different fertilization strategies on soil health, SOC, and soil pH; (ii) evaluate biogas slurry impact on soybean productivity in black soil conditions; (iii) analyze the biogas slurry influence on microbial communities dynamics.

## 2 Materials and methods

### 2.1 Site description

This study was conducted in northeastern China, within Heilongjiang Province. The experiment was established at Harbin’s National Modern Agriculture Demonstration Park, managed by the Heilongjiang Academy of Agricultural Sciences (45.73°N, 126.66°E). The region experiences a mid-temperate continental monsoon climate, with long, cold winters and relatively short summers. Mean annual air temperature is approximately 3.5°C, and the mean annual precipitation is around 524 mm, most of which falls from between June and September. The soil classification is Mollisol, commonly referred to as black soil. The soil had a sandy clay loam texture, containing 16.58 g/kg organic carbon, and 1.86 g/kg total nitrogen.

### 2.2 Experimental design

A controlled pot experiment was set up using pots with standardized dimensions with an upper diameter of 28 cm, a lower diameter of 18 cm, and a height of 26 cm. Each pot was filled with 10 kg of air-dried black soil, leaving a 10 cm margin from the top. Small drainage holes were made at the base of each pot. Uniform soil conditions were maintained across all pots. Soybeans were sown directly into the pots, and after emergence, the seedlings were thinned to four uniform plants per pot. The experiment was initiated in July 2024 and observed for approximately 4 months until soybean maturity. A randomized complete block design was employed to minimize spatial variability. Pots were arranged randomly within the experimental area and repositioned periodically during the experiment to minimize positional effects. Irrigation was applied based on the plant requirements throughout the experimental duration to maintain adequate and consistent soil moisture conditions, and plant growth was regularly monitored. Four treatments were tested with three replications each: (1) no fertilizer application (control); (2) Chemical Fertilizers (150 kg N/ha, 80 kg P/ha, 40 kg K/ha); (3) Biogas Slurry (150 kg N/ha); (4) Biogas Slurry + chemical fertilizers. The biogas slurry derived from cow dung was sourced from the Key Laboratory of Agricultural Renewable Resources Utilization Technology and Equipment in Cold Areas of Northeast Agricultural University. It contains 318.41 g/kg soil organic carbon and 24.1 g/kg total nitrogen, and heavy metals including Cu (27.9 mg/kg), Zn (90.68 mg/kg), Pb (9.46 mg/kg), and Cd (0.3 mg/kg).

At maturity, yield components, including the number of pods per plant, seeds per plant, and seeds per pot, were recorded. Grain yield was calculated by harvesting all plants in each pot.

### 2.3 Soil sampling and laboratory analysis

Representative black soil samples (0–20 cm depth) were collected using standard method (Feng et al., 2024). Samples were air-dried, passed through a 6 mm sieve, and homogenized using a 2 mm screen for analysis or storage (Bremner and Mulvaney, 1982). SOC was measured using the potassium dichromate oxidation method with ferrous sulfate titration (Walkley and Black, 1934). TN was quantified by the Kjeldahl digestion procedure (Bremner, 1960), while TP was determined by the molybdenum antimony colorimetric method. Soil pH was measured in a 2.5:1 water to soil suspension using a pH meter (Han et al., 2018). Microbial biomass carbon (MBC), microbial biomass nitrogen (MBN), and microbial biomass phosphorus (MBP) were assessed by the chloroform fumigation-incubation technique. The microbial biomass was extracted with K2SO4 following the method described by Brookes et al. (1985) (Vance et al., 1987). Heavy metal concentrations of Cu, Cd, Zn, and Pb were analyzed using inductively coupled plasma mass spectrometry (ICP-MS; Agilent 7800, United States) after acid digestion following standard methods (Hou et al., 2018; Tian et al., 2025). Prior to ICP-MS analysis, soil samples were digested using a mixture of concentrated nitric acid (HNO_3_) and perchloric acid (HClO_4_) under controlled heating conditions following standard protocols.

Plant physiological data (chlorophyll content and leaf nitrogen concentration) were collected at 30, 60, and 90 days after planting using a SPAD-502 Chlorophyll Meter (Hangzhou Meinan Instruments, China), providing a rapid, non-destructive estimate of photosynthetic potential and nutrient status.

### 2.4 DNA Extraction and PCR amplification

Soil genomic DNA was isolated using the FastDNA SPIN Kit for Soil (MP Biomedicals, United States) following the protocol supplied by the manufacturer. The yield and purity of extracted DNA were checked with a NanoDrop spectrophotometer (Thermo Fisher Scientific, United States). To characterize bacterial communities, the V3–V4 region of the 16S rRNA gene was amplified with primers 338F (5′–ACTCCTACGGGAGGCAGCAG–3′) and 806R (5′–GGACTACHVGGGTWTCTAAT–3′). Fungal communities were assessed by amplifying the ITS2 region of the rDNA using primers ITS1F (5′–CTTGGTCATTTAGAGGAAGTAA–3′) and ITS2R (5′–GCTGCGTTCTTCATCGATGC–3′). Each PCR was performed in a 20 μL reaction volume containing 10 ng of template DNA, 0.8 μM of each primer, and 10 μL of 2 × PCR Master Mix (Li Y. et al., 2022). PCR amplification was performed using a high-fidelity DNA polymerase, and amplification cycles were carefully controlled to ensure consistency across samples. Negative controls were included to detect potential contamination.

### 2.5 Sequencing and data processing

PCR products were purified using the AxyPrep DNA Gel Extraction Kit (Axygen Biosciences, United States) and quantified using the QuantiFluor™ ST Blue Fluorescence Quantification System (Promega, United States). The quality of sequencing libraries was assessed using an Agilent Bioanalyzer to verify fragment size distribution and the absence of adapter contamination. Library concentration was quantified using a fluorescence-based method prior to sequencing. High-throughput sequencing was carried out on an Illumina MiSeq platform (Illumina, United States). Raw sequence reads were quality-checked with FastQC, merged using FLASH, and analyzed using MOTHUR v.1.34.1. Low-quality reads (Q < 20), ambiguous sequences, and chimeric reads were removed during quality filtering. Sequences were demultiplexed based on barcode information prior to downstream analysis. Taxonomic classification was elaborated with the Silva 16S rRNA reference database, whereas fungal (ITS) reads were classified against the UNITE database (Ren et al., 2020). Beta diversity analysis was performed using Bray-Curtis dissimilarity, and principal coordinate analysis (PCoA) was used to visualize differences in microbial community structure among treatments. Heatmaps and Venn diagrams were generated to illustrate differences in taxonomic composition and shared or unique OTUs across treatments. The microbial analysis was designed to assess treatment-driven differences in community composition and structure, in line with the main objectives of the study.

### 2.6 Statistical analysis

The statistical analyses were implemented with IBM SPSS Statistics 27 (IBM Corp., Armonk, NY, United States). A one-way analysis of variance (ANOVA) was applied to test treatment effects on soil chemical properties (SOC, TN, TP, pH), microbial biomass (MBC, MBN, MBP), yield components, and nitrogen transformation rates. Normality of residuals and homogeneity of variance were assessed prior to ANOVA to ensure that the assumptions of the analysis were met. Homogeneity of variances was verified, and Tukey’s Honest Significant Difference (HSD) test was included for multiple comparisons when appropriate. For time-dependent variables (leaf nitrogen concentration, chlorophyll content, and nitrogen mineralization), a general linear model was used. Pearson’s correlation analysis was performed using metan package in R (version 4.4.2). The results are reported as means with standard deviation (SD). Graphs were built with SigmaPlot version 15.0 to visualize treatment effects and temporal trends.

## 3 Results

### 3.1 Impact of biogas slurry application on black soil nutrients

Fertilizer application had a significant effect on soil nutrient levels (*P* < 0.05). Compared to the unfertilized control, all fertilizer treatments significantly increased SOC, TN, and TP. The highest increases in SOC (8.97 g/kg) and TN (1.30 g/kg) were observed in the biogas slurry treatment. The combined treatment of biogas slurry and chemical fertilizer (mixed fertilizer) resulted in higher SOC (8.22 g/kg) than the chemical fertilizer treatment alone (8.05 g/kg), although the difference in TN between treatments was not statistically significant. For TP, the chemical fertilizer treatment showed a significant increase (0.92 g/kg) compared to the control (Table 1).

**TABLE 1:** Effect of fertilizer application on soil organic carbon (SOC), total nitrogen (TN), and total phosphorus (TP) content in black soil.

Treatment	SOC (g/kg)	TN (g/kg)	TP (g/kg)
Unfertilized control (CK)	6.56 ± 0.04a	1.17 ± 0.01a	0.87 ± 0.01ab
Chemical fertilizer (N)	8.05 ± 0.01b	1.23 ± 0.11a	0.92 ± 0.03b
Biogas slurry (BS)	8.97 ± 0.26c	1.3 ± 0.11a	0.87 ± 0.07ab
Mixed fertilizer (BS+N)	8.22 ± 0.16b	1.21 ± 0.06a	0.8 ± 0.06a
	*P* < 0.001	*P* > 0.05	*P* < 0.02

Mixed fertilizer: Biogas slurry and chemical fertilizer combination. Data are presented as mean values for different treatments. Different lowercase letters indicate significant differences among treatments (*P* < 0.05).

**FIGURE 1:**
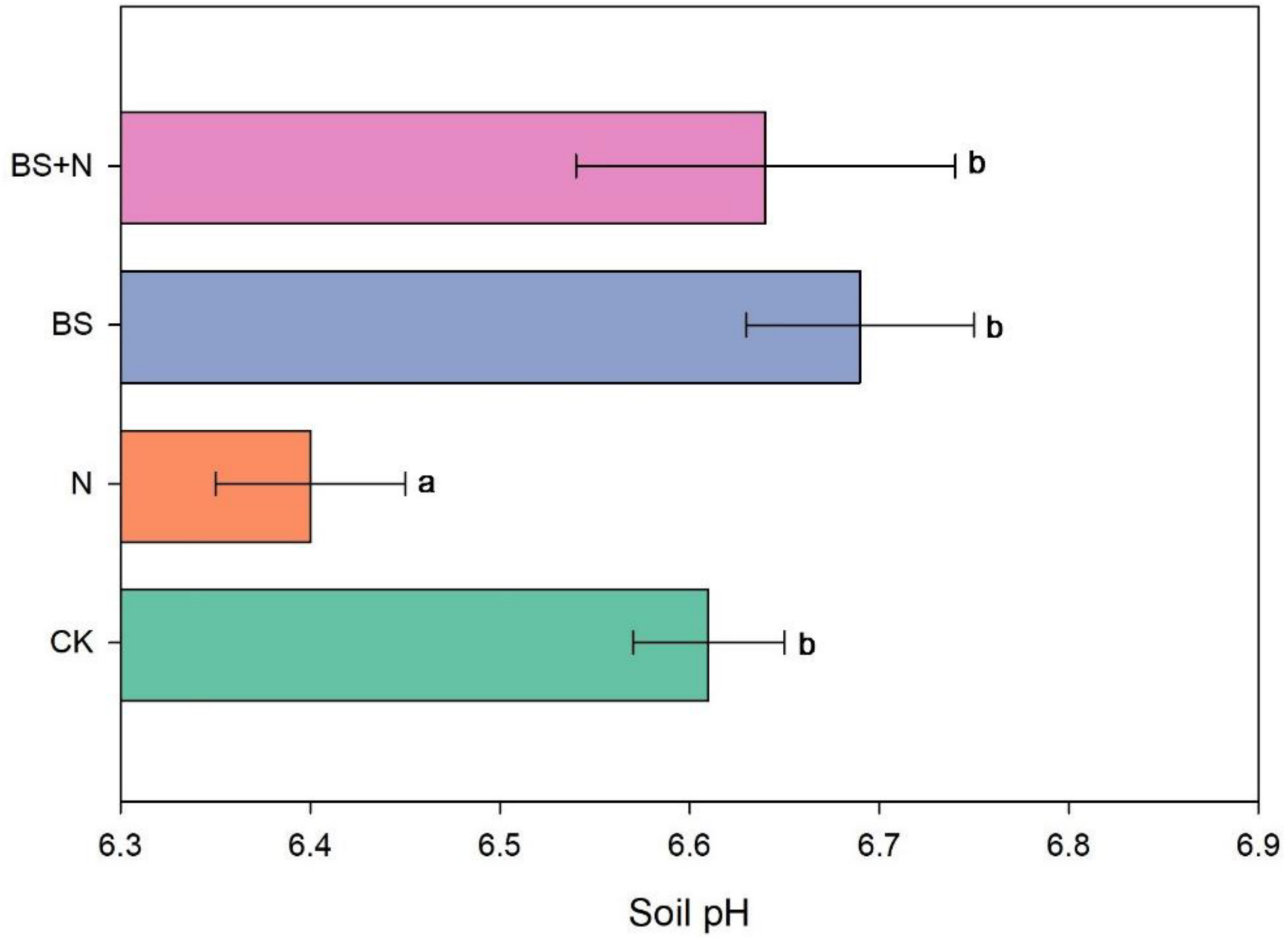
Changes in black soil pH under different fertilizer treatments. Data represent mean values; error bars indicate standard deviation. The results highlight the acidifying effect of chemical fertilizers and the pH-buffering capacity of biogas slurry applications. Different lowercase letters above the columns represent statistical significance among treatments at *P* < 0.05. CK, unfertilized; N, Chemical fertilizer; BS, Biogas slurry; BS+N, mixed fertilizer (biogas slurry and chemical fertilizer).

Our study revealed that black soil pH ranged from 6.40 to 6.69 across the different treatments (Figure 1). Chemical fertilizer application led to a noticeable acidification of the soil, decreasing the pH from 6.61 in the unfertilized control to 6.40. In contrast, treatments involving biogas slurry exhibited a buffering effect, mitigating soil acidification and slightly increasing pH levels. The biogas slurry resulted in the highest pH value of 6.69, while the mixed fertilizer treatment also increased the soil pH to 6.64 (Figure 1).

### 3.2 Influence of biogas slurry on microbial biomass carbon, microbial biomass nitrogen, and microbial biomass phosphorus in black soil

**FIGURE 2:**
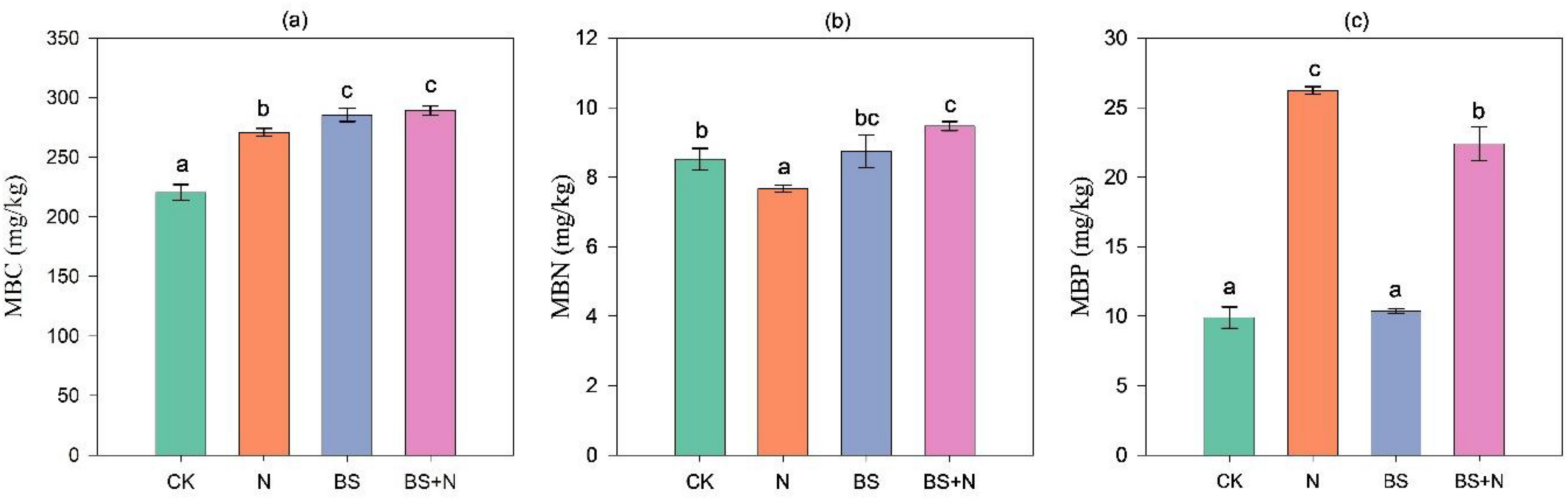
Changes in microbial biomass carbon **(a)**, microbial biomass nitrogen **(b)**, and microbial biomass phosphorus **(c)** in black soil under different fertilizer applications. Data represent mean values; error bars indicate standard deviation. Different lowercase letters above the columns represent statistical significance among treatments at *P* < 0.05. CK: unfertilized, N, Chemical fertilizer; BS, Biogas slurry; BS+N, mixed fertilizer (biogas slurry and chemical fertilizer).

Our study showed that fertilizer application significantly increased MBC compared to the unfertilized control (Figure 2). Among the treatments, mixed fertilizer exhibited the highest MBC (289.23 mg/kg), followed closely by biogas slurry (285.65 mg/kg). Chemical N fertilizer had a smaller increase in MBC (270.92 mg/kg). Regarding MBN, treatments containing biogas slurry significantly enhanced MBN levels. The highest MBN (9.47 mg/kg) was observed in the treatment combining biogas slurry with chemical N fertilizer. Biogas slurry alone increased MBN to 8.74 mg/kg. In contrast, chemical N fertilizer alone significantly reduced MBN (7.67 mg/kg; *P* < 0.05) compared to the unfertilized soil. For MBPs, chemical N fertilizers resulted in the highest increase (26.24 mg/kg), followed by a combination of slurry biogas and chemical fertilizers (22.41 mg/kg). Biogas slurry alone led to a small increase in MBP (10.37 mg/kg) compared to the unfertilized control (Figure 2).

### 3.3 Influence of biogas slurry on heavy metal content in black soil

Fertilizer treatments had a significant effect on soil heavy metal content, particularly for Cu and Cd (*P* < 0.05). Compared to the unfertilized control, biogas slurry application significantly increased Cu concentration in the soil (Table 2). The highest Cu level (38.66mg/kg) was observed in the BS treatment, which was significantly higher than in the control (17.47mg/kg), chemical fertilizer (18.07mg/kg), and mixed fertilizer (13.5mg/kg) treatments (*P* < 0.001). In contrast, the mixed fertilizer treatment significantly reduced Cu levels compared to BS alone. Zn concentrations ranged from 38.82mg/kg (control) to 41.71mg/kg (mixed fertilizer), but no significant changes were found among treatments (*P* > 0.05), indicating that biogas slurry application did not affect Zn accumulation in the short term. Pb content also remained statistically unchanged across treatments (*P* > 0.05), with values ranging from 18.51mg/kg in the control to 21.99mg/kg in the N treatment. For Cd, a slight but statistically significant increase was detected in both the BS (0.13mg/kg) and N (0.13mg/kg) treatments compared to the control (0.11mg/kg) and the mixed fertilizer (0.11mg/kg) (*P* < 0.05) (Table 2).

**TABLE 2:** Effect of fertilizer application on copper (Cu), zinc (Zn), lead (Pb), and cadmium (Cd) content in black soil.

Treatment	Cu (mg/kg)	Zn (mg/kg)	Pb (mg/kg)	Cd (mg/kg)
Unfertilized control (CK)	17.47 ± 0.50a	38.82 ± 2.05a	18.51 ± 4.47a	0.11 ± 0.01a
Chemical fertilizer (N)	18.07 ± 0.99a	40.61 ± 3.13a	21.99 ± 1.733a	0.13 ± 0.00b
Biogas slurry (BS)	38.66 ± 9.58b	39.49 ± 0.47a	21.39 ± 0.32a	0.13 ± 0.01b
Mixed fertilizer (BS+N)	13.5 ± 0.05a	41.71 ± 4.06a	19.58 ± 0.03a	0.11 ± 0.05a
	*P* < 0.001	*P* > 0.05	*P* > 0.05	*P* < 0.05

Mixed fertilizer: Biogas slurry and chemical fertilizer combination. Different lowercase letters indicate significant differences among treatments (*P* < 0.05).

### 3.4 Influence of biogas slurry on plant physiological development

Our study showed that fertilizer application significantly influenced chlorophyll content and N concentration in the leaves at 90 days after planting (Table 3). At this stage, the highest chlorophyll content was observed in plants treated with chemical fertilizer (31.95 SPAD), followed closely by mixed fertilizer (31.8 SPAD). In contrast, biogas slurry alone resulted in the lowest chlorophyll content among the fertilized treatments (28.11 SPAD). No significant differences in chlorophyll content were observed among treatments at 30 and 60 days. Similarly, leaf N content at 90 days significantly increased with mixed fertilizers (12.92 mg/g), as well as with chemical fertilizer alone (12.9 mg/g), compared to unfertilized control (12.19 mg/g). However, biogas slurry alone led to a significant reduction in leaf N content (11.53 mg/g). As with chlorophyll, no significant effects on leaf N content were observed at 30 and 60 days (Table 3).

**TABLE 3:** Effect of fertilizer application on chlorophyll content and leaf nitrogen (N) content at different periods.

Treatment	Chlorophyll (SPAD)			leaf N content (mg/g)		
	30 Days	60 Days	90 Days	30 Days	60 Days	90 Days
Unfertilized control (CK)	32.83 ± 0.36a	34.39 ± 0.7a	30.15 ± 0.83ab	13.03 ± 0.10a	13.53 ± 0.22a	12.19 ± 0.26b
Chemical fertilizer (N)	33.22 ± 0.81a	34.07 ± 0.97a	31.95 ± 0.37b	13.16 ± 0.26a	13.43 ± 0.31a	12.9 ± 0.14c
Biogas slurry (BS)	30.82 ± 2.31a	33.22 ± 0.63a	28.11 ± 0.97a	12.6 ± 0.43a	13.16 ± 0.2a	11.53 ± 12.92a
Mixed fertilizer (BS+N)	32.12 ± 0.71a	34.89 ± 0.34a	31.8 ± 1.01b	12.81 ± 0.23a	13.69 ± 0.11a	12.92 ± 0.11c
Anova	*P* > 0.05	*P* > 0.05	*P* < 0.001	*P* > 0.05	*P* > 0.05	*P* < 0.001

Mixed fertilizer: Biogas slurry and chemical fertilizer combination. Different lowercase letters indicate significant differences among treatments (*P* < 0.05).

### 3.5 Impact of biogas slurry on soybean productivity

**FIGURE 3:**
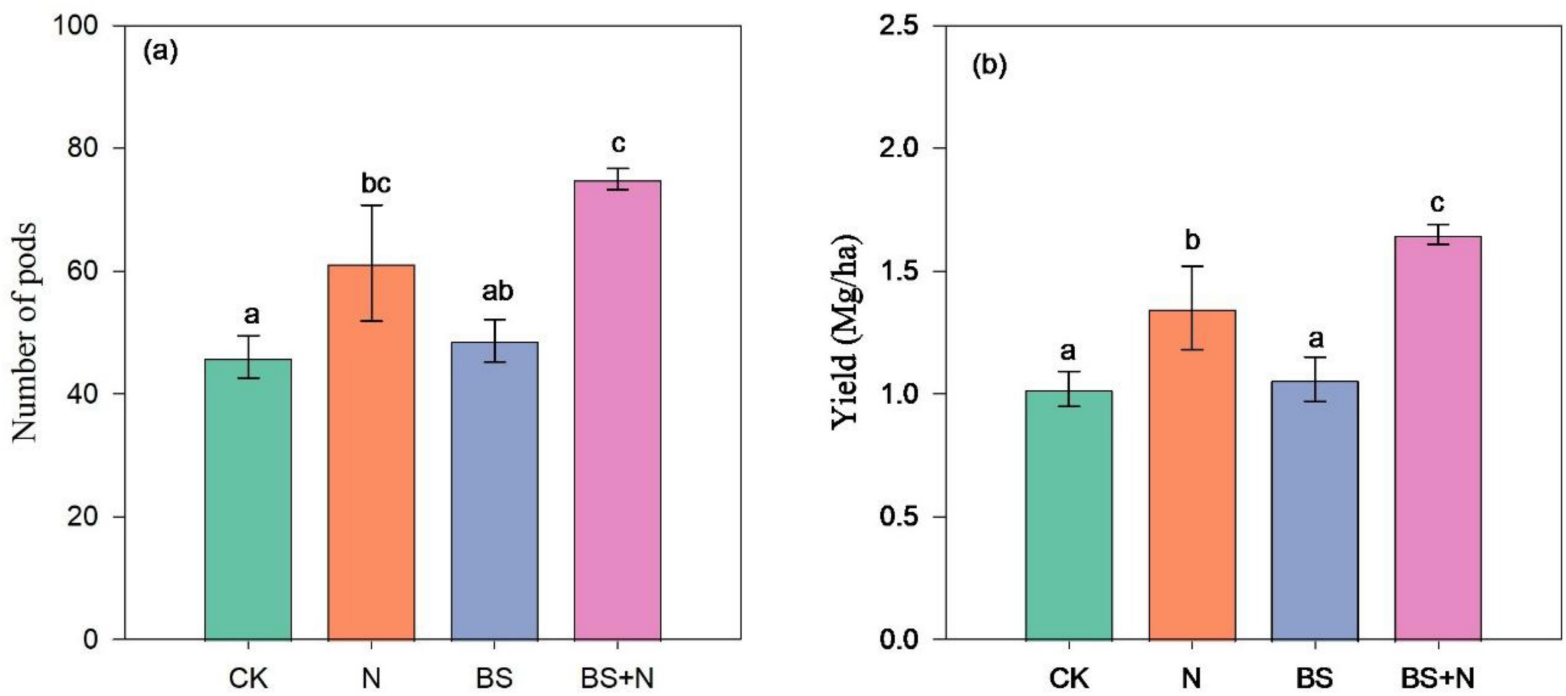
Effect of fertilizer application on **(a)** the number of pods per plant and **(b)** soybean grain yield. Different lowercase letters above the columns represent statistical significance among treatments at *P* < 0.05. CK: unfertilized, N, Chemical fertilizer; BS, Biogas slurry; BS+N, mixed fertilizer (biogas slurry and chemical fertilizer).

Our study showed that fertilizer application significantly increased the number of pods per pot compared to unfertilized soil (Figure 3). The highest average pod number was recorded with mixed fertilizer (75 pods), followed by chemical fertilizer alone (61.33 pods), and biogas slurry alone (48.67 pods). In terms of grain yield, the highest soybean yield was achieved with the combined application of biogas slurry and chemical N, reaching 1.65 Mg/ha. This was followed by chemical fertilizer alone, which also significantly improved yield (1.35 Mg/ha) compared to the unfertilized control. The lowest yields were recorded for biogas slurry alone (1.06 Mg/ha) and the control (CK) treatment (1.02 Mg/ha) (Figure 3).

### 3.6 Impact of fertilizers on the correlation between soybean yield and black soil nutrients

**FIGURE 4:**
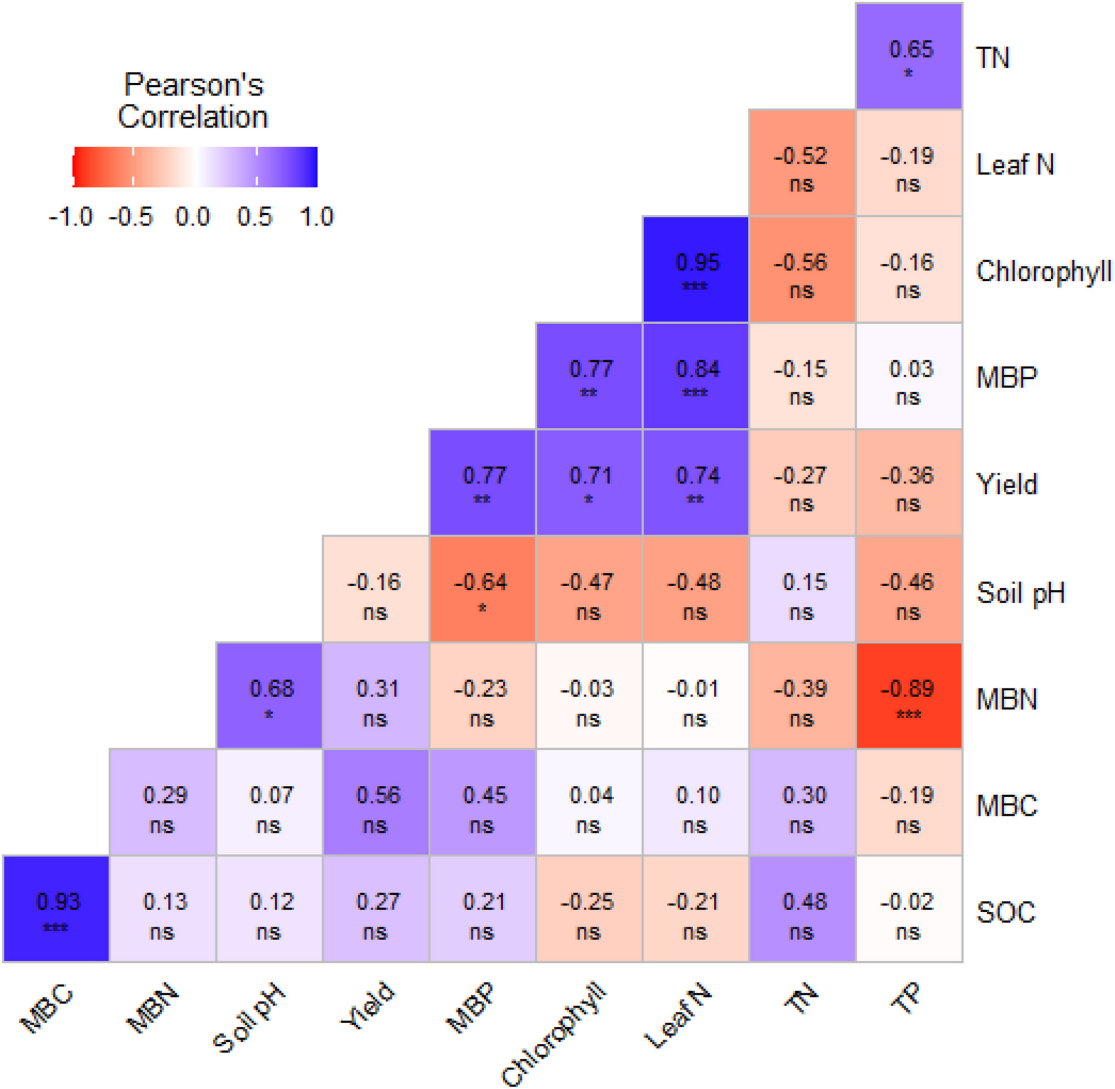
Correlation matrix showing relationships between soybean grain yield and black soil nutrient indicators. significance levels: ns non-significance *p* > 0.05, * significance at *p* < 0.05, ** significance at *p* < 0.01, *** significance at *p* < 0.001.

The correlation analysis showed a strong and significant positive relationship (*p* < 0.001) between MBC and SOC (*r* = 0.93), suggesting that increased MBC activity is associated with increased SOC (Figure 4). A highly significant negative correlation was found between MBN and TP (*r* = -0.89), indicating that higher MBN may be linked to reduced phosphorus availability. Soil pH indicated a moderate positive correlation with MBN (*r* = 0.68), implying that higher MBN may contribute to increased soil pH. In contrast, soil pH was negatively correlated with MBP (*r* = -0.64). A significant positive correlation was found between grain yield and MBP (*r* = 0.77), chlorophyll content (*r* = 0.71), and leaf N content (*r* = 0.74). Furthermore, chlorophyll content was strongly correlated with both leaf N content (*r* = 0.95) and MBP (*r* = 0.77) (Figure 4).

### 3.7 Impact of fertilizer application on bacterial and fungal communities

**FIGURE 5:**
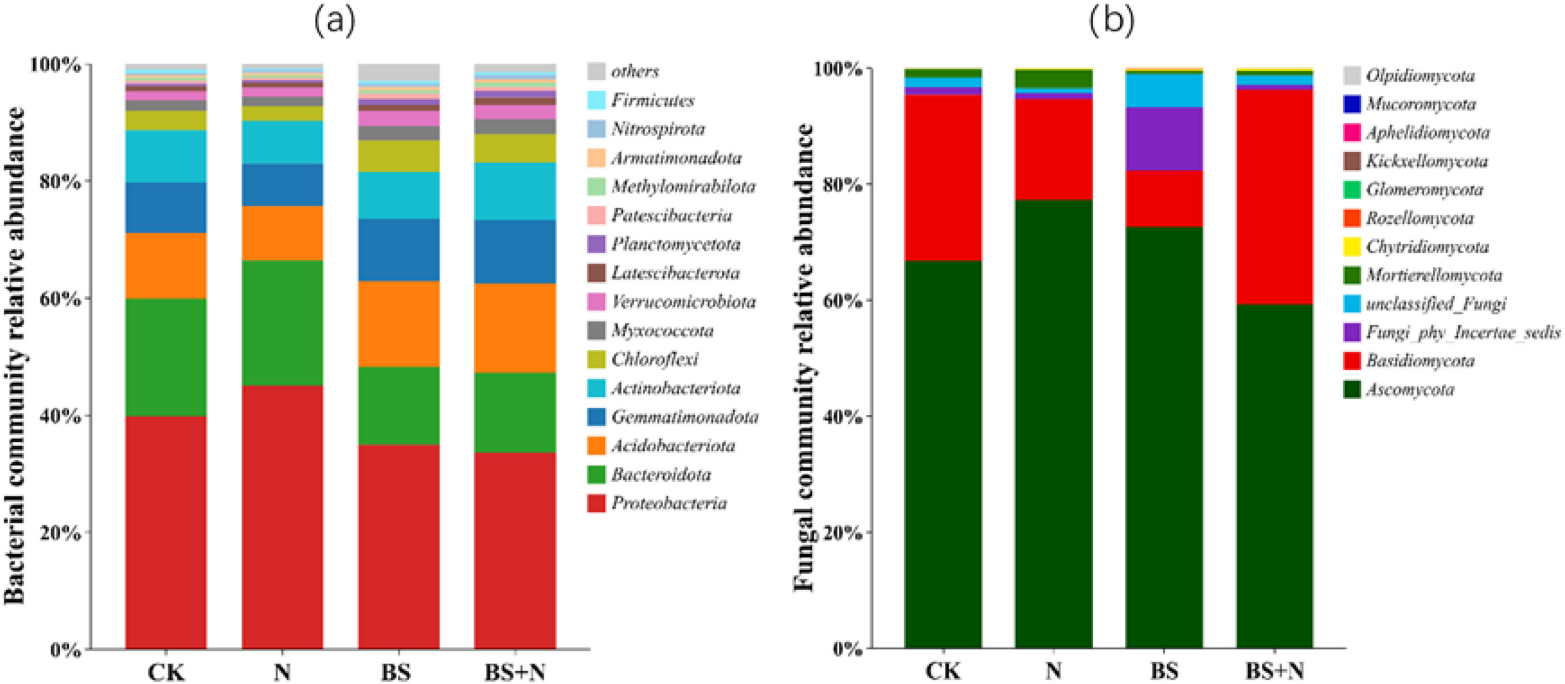
Relative abundance of dominant soil bacterial **(a)** and fungal **(b)** phyla following fertilizer application. CK, unfertilized; N, Chemical fertilizer; BS, Biogas slurry; BS+N, mixed fertilizer (biogas slurry and chemical fertilizer).

Analysis of the relative abundance of the bacterial communities revealed that the most dominant phyla were Proteobacteria (33.67–45.07%), Bacteroidota (13.29–21.36%), Acidobacteriota (9.26–15.16%), Gemmatimonadota (7.35–10.89%), and Actinobacteriota (7.23–9.75%) (Figure 5). Biogas slurry and mixed fertilizers enhanced the relative abundance of *Acidobacteriota*, *Gemmatimonadota*, and *Chloroflexi*. In contrast, the abundance of *Proteobacteria* and *Bacteroidota* decreased with the addition of biogas slurry and mixed fertilizers.

For the fungal community, the dominant phyla were Ascomycota (59.25–77.29%), Basidiomycota (9.65–37.05%), and Fungi_phy_Incertae_sedis (0.76–10.96%). The application of biogas slurry increased *Ascomycota* and *Fungi_phy_Incertae_sedis*, and decreased *Basidiomycota*, compared to the unfertilized control. In contrast, the use of mixed fertilizer reduced the relative abundance of *Ascomycota* while increasing that of *Basidiomycota* (Figure 5).

**FIGURE 6:**
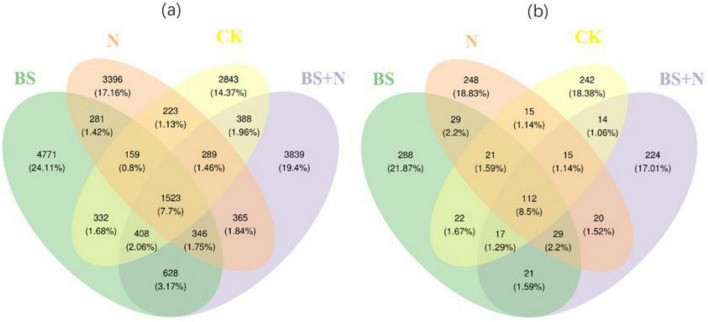
Venn diagrams showing the shared and unique OTUs of bacterial **(a)** and fungal **(b)** communities following fertilizer application. CK, unfertilized; N, Chemical fertilizer; BS, Biogas slurry; BS+N, mixed fertilizer (biogas slurry and chemical fertilizer).

Soils treated with biogas slurry exhibited the highest number of unique bacterial OTUs (4771), followed by mixed and chemical fertilizer treatments with 3839 and 3396 unique OTUs, respectively. For the fungal community, the highest number of unique OTUs was also observed under biogas slurry treatment (288), followed by chemical fertilizer (248), whereas mixed fertilizer application resulted in the lowest number (224) (Figure 6).

### 3.8 Impact of fertilization on microbial community structure

**FIGURE 7:**
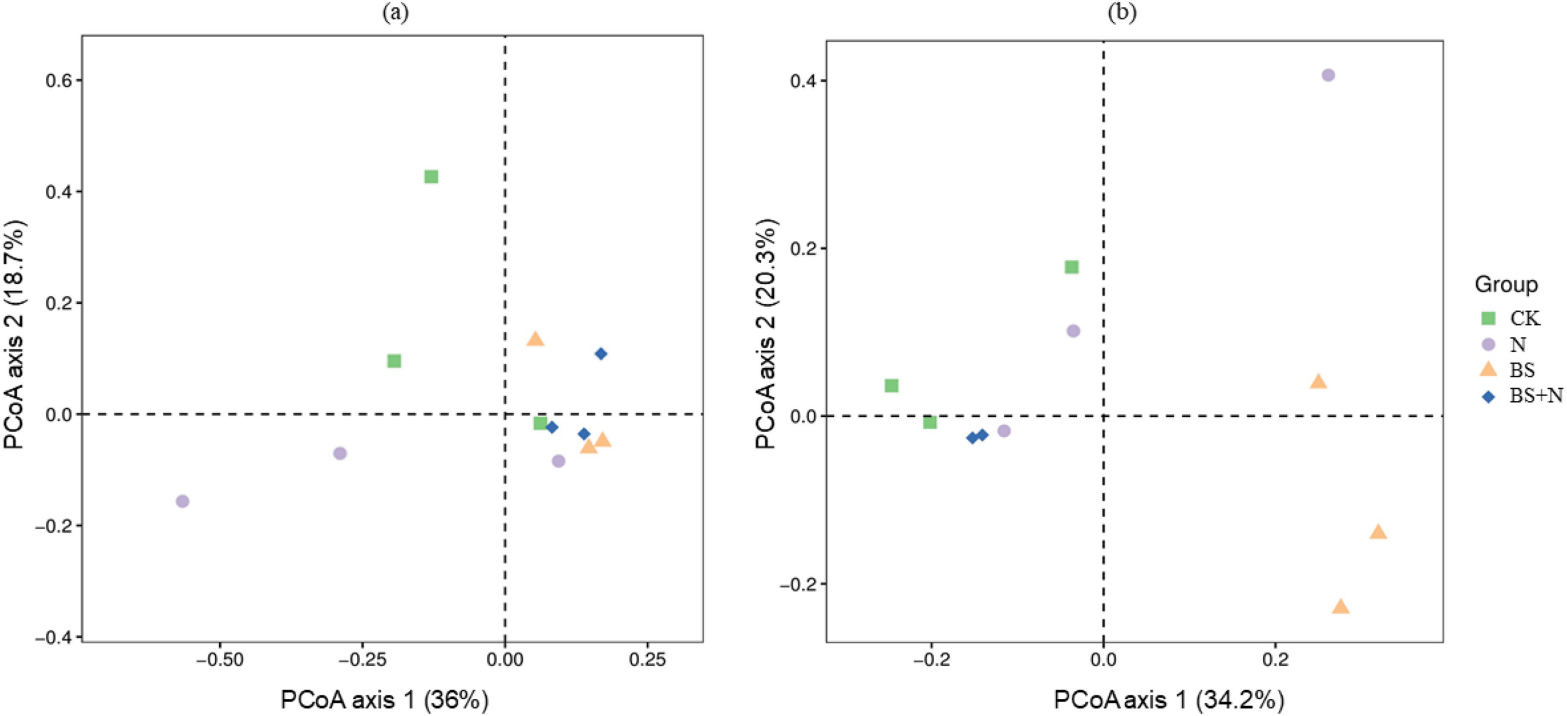
PCoA of soil Bacterial **(a)** and Fungal **(b)** communities under different fertilization treatments. CK, unfertilized; N, Chemical fertilizer; BS, Biogas slurry; BS+N, mixed fertilizer (biogas slurry and chemical fertilizer).

PCoA analysis revealed distinct patterns in microbial community composition across treatments (Figure 7). For the bacterial community, the first two axes explained 36.0 and 18.7% of the total variation, respectively. Samples showed partial separation among treatments, with some overlap observed among fertilized treatments, indicating a moderate effect of fertilization on bacterial community structure (Figure 7a). In contrast, the fungal community exhibited a clearer separation among treatments, with the first two axes explaining 34.2 and 20.3% of the total variation, respectively (Figure 7b). In particular, the biogas slurry (BS) treatment was distinctly separated from other treatments, suggesting an influence of fertilization on fungal community composition.

**FIGURE 8:**
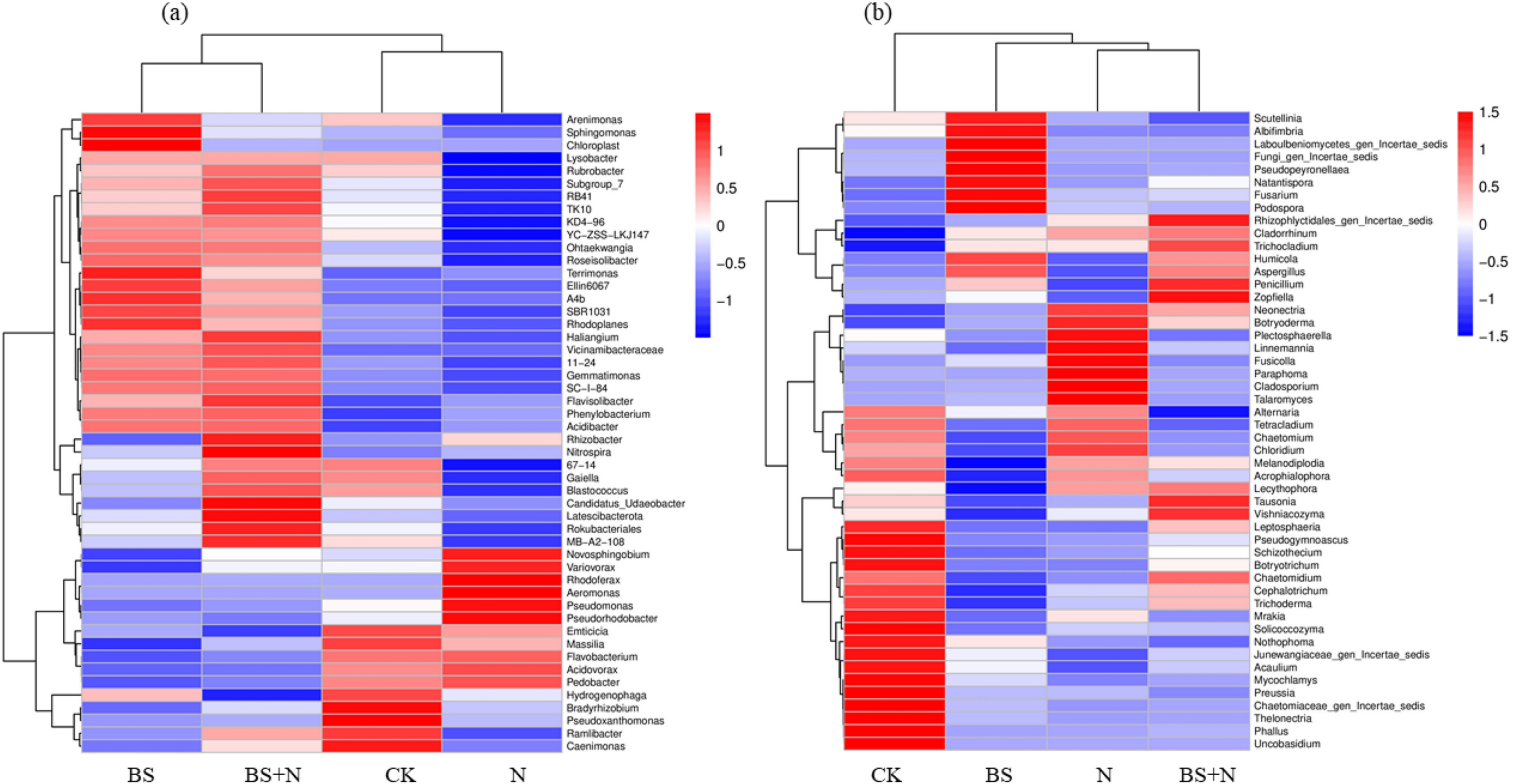
Heatmap of soil bacterial **(a)** and fungal **(b)** communities under different fertilization treatments. CK, unfertilized control; N, chemical fertilizer; BS, biogas slurry; BS+N, mixed fertilizer (biogas slurry and chemical fertilizer).

The heatmap analysis further supported these observations by showing distinct patterns in the relative abundance of dominant microbial taxa across treatments (Figure 8). For the bacterial community (Figure 8a), variations in taxa abundance were observed among treatments, with clustering analysis indicating differences between the control (CK) and fertilized treatments. However, the overall patterns showed moderate differentiation, with several taxa shared across treatments. The fungal community (Figure 8b) displayed more pronounced compositional shifts, with clear clustering of treatments. Notably, BS and BS+N treatments exhibited distinct abundance patterns compared with CK and N treatments, indicating a stronger restructuring of fungal communities in response to fertilization.

## 4 Discussion

### 4.1 Impact of biogas slurry on SOC, soil pH regulation, and microbial biomass

The increase in SOC and TN under biogas slurry treatment (Table 1) indicates its potential role as a nutrient source. Unlike mineral fertilizers, which supply only inorganic nutrients, biogas slurry introduces complex organic substrates, including labile carbon compounds, humic precursors, and bioavailable nitrogen that activate microbial metabolism and stimulate carbon turnover (Feng et al., 2024; Li P. C. et al., 2025). These processes align with findings from Bohoussou et al. (2023), who highlighted how organic amendments improve long-term carbon sequestration potential by promoting microbial residue formation (Wang et al., 2025). The observed correlation between SOC and MBC highlights the coupling between substrate availability and microbial proliferation, where microbial activity becomes a key regulator of carbon stabilization in amended black soils (Cai et al., 2021). These results are consistent with recent studies reporting that digestate- or slurry-based organic amendments increase SOC and microbial biomass more effectively than mineral fertilizers because of carbon substrates and available nutrients for microbial growth (Cucina et al., 2025; Engedal et al., 2025). The response observed in this study suggests that black soils of Northeast China may particularly benefit from biogas slurry inputs, likely due to their high native organic matter and buffering capacity, which can favor the microbial processing of added organic matter.

In contrast, the exclusive use of chemical fertilizers provided inorganic nutrients that accelerated plant growth (Bohoussou et al., 2025) but failed to enhance soil carbon pools due to the absence of organic carbon input (Bohoussou et al., 2026). As mineral N stimulates root uptake but not organic carbon input, long-term use often leads to carbon depletion and a decline in microbial-derived organic matter (Liu et al., 2025). By comparison, the mixed treatment maintained intermediate SOC levels, balancing rapid nutrient supply with longer-term carbon input. This balance minimizes nutrient leaching losses while supporting continuous microbial activity throughout the crop cycle (Bohoussou et al., 2022). This pattern is also in agreement with recent reports showing that integrated organic-mineral fertilization generally performs better than mineral fertilization alone because it combines rapid nutrient supply with improved carbon input and microbial stimulation (Liu et al., 2026; Uddin et al., 2025).

Soil acidification under mineral fertilization was consistent with proton release during nitrification and leaching of basic cations, processes known to disrupt microbial equilibrium and nutrient cycling (He et al., 2023). In contrast, the alkaline nature of biogas slurry, rich in bicarbonates and base cations such as Ca^2+^ and Mg^2+^, offsets these acidifying effects. Its buffering capacity restored near-neutral pH conditions conducive to microbial growth and enzymatic activity (Zhang et al., 2021; Wang et al., 2023). This neutralization not only mitigates nutrient depletion but also stabilizes organic matter and supports microbial nutrient transformations (Yin et al., 2025), suggesting that biogas slurry may function as both a carbon input and soil conditioner under the conditions tested (Mukhtiar et al., 2024). Similar pH-buffering effects of digestate and other organic amendments have been reported in intensively fertilized cropping systems, although the magnitude of the response may vary depending on soil texture, initial pH, and amendment composition (Liu et al., 2026).

The elevated MBC and MBN under biogas slurry and mixed fertilizer suggest improved microbial resource quality and stoichiometric balance. Biogas slurry supplies a broad spectrum of easily degradable organic compounds, such as amino acids, simple carbohydrates, and ammonium-N, that provide microbes with both energy sources and essential nutrients (Kumar et al., 2023). When these labile substrates enter the soil system, microbial communities respond rapidly through increased biomass synthesis and metabolic activity (Yadav et al., 2023). Moreover, the balanced supply of carbon and nitrogen in biogas slurry supports efficient microbial metabolism, reducing stoichiometric constraints commonly observed under mineral fertilizer regimes where nitrogen is abundant but organic carbon is limited (Yin et al., 2025). By contrast, chemical fertilizer alone enhanced MBP, reflecting transient microbial phosphorus assimilation from soluble inorganic sources (Alori et al., 2017). Without sufficient carbon co-supply, however, this response is unsustainable, leading to microbial stress and reduced biomass persistence (Tian et al., 2021). The observed relationships among MBN, pH, and MBP further underline the role of biogas slurry in modulating soil biochemical equilibrium, where balanced carbon and nutrient inputs favor microbial growth while preventing excessive acidification or nutrient immobilization (da Silva et al., 2023). Our findings are in line with recent studies showing that organic amendments stimulate microbial biomass relative to mineral fertilizers because they alleviate carbon limitation and improve microbial stoichiometric balance (Mao et al., 2024). The response observed here is especially relevant for black soils, where maintaining active microbial biomass is critical for preserving organic matter turnover and reducing fertility decline under intensive cultivation. Overall, these findings indicate that biogas slurry may contribute to improved soil carbon dynamics and microbial activity, which are consistent with the observed changes in soil properties.

### 4.2 Biogas slurry induced changes in heavy metal accumulation in black soil

The significant increase in soil Cu content observed under biogas slurry treatment (Table 2) reflects both the metal load in the biogas slurry and the strong affinity of Cu for organic ligands introduced with it. In black soils, which are rich in clay minerals and humic substances, these ligands can complex Cu into relatively stable organometallic forms. Over time, however, these complexes can become immobilized through sorption onto clay minerals and Fe/Mn oxides, which are abundant in black soils (Li Q. et al., 2022). This duality, characterized by temporary solubilization followed by stabilization, suggests that Cu introduced by biogas slurry may initially circulate in the soil solution before integrating into stable organomineral fractions, thereby becoming more available in the soil solution (Cela-Dablanca et al., 2022). The lower Cu levels under mixed fertilizer compared to biogas slurry alone suggest a dilution or immobilization effect, likely due to phosphate-induced precipitation or ion competition triggered by the mineral fertilizer component. This dilution or immobilization could reflect antagonistic interactions, where the addition of nitrates or phosphates influences Cu sorption behavior or precipitation (Kim and Park, 2024). Comparable increases in Cu following slurry or digestate application have been reported in other agricultural soils, but the extent of accumulation depends strongly on soil organic matter content and metal sorption capacity, which are particularly high in black soils (Yin et al., 2026).

Slight increases in Cd concentrations under biogas slurry and N treatments are of concern because Cd is highly mobile and generally weakly retained on soil colloids compared with other trace metals (Alengebawy et al., 2021). Competition with Ca^2+^, Mg^2+^, Zn^2+^ and other cations supplied by the slurry can displace Cd from exchange sites, increasing its presence in the soil solution and potentially its bioavailability (Yu et al., 2023). In addition, localized acidification associated with ammonium nitrification in mineral N treatments can further enhance Cd solubility and weaken its association with organic matter and clay minerals (Li Q. et al., 2025). Even though the Cd concentrations measured in this study remain below regulatory thresholds, the combination of high mobility, efficient plant uptake, and the cumulative nature of repeated applications means that Cd could gradually accumulate in the plough layer over time. This risk is particularly relevant for food crops such as soybean, where edible tissues may act as a pathway for Cd transfer into the human diet (Rigby and Smith, 2020). Consequently, long-term fertilization strategies involving biogas slurry should be accompanied by regular monitoring of Cd inputs and soil stocks, as well as careful control of application rates and frequency when scaling up its use in food production systems. In contrast, the unchanged Zn and Pb levels suggest strong binding of these metals to clay minerals and stable organic matter under near-neutral pH conditions. The absence of short-term changes in Zn and Pb indicates that the application rate used in this experiment is environmentally safe, but continued monitoring is essential to assess cumulative impacts. Overall, the results indicate that biogas slurry amendments introduce both nutrient and trace element inputs, requiring careful management to balance agronomic benefits and potential environmental risks. Recent studies on organic fertilizers have similarly emphasized that Cd requires particular attention because of its greater mobility and plant transfer potential relative to Zn and Pb (Liu et al., 2024). In this context, our results reinforce that evaluating the agronomic benefits of biogas slurry should be accompanied by trace metal monitoring, especially in food-producing systems. It should be noted that the present study evaluated only total metal concentrations in soil. Therefore, the results cannot directly reflect metal bioavailability, plant uptake, or ecological risk, which would require additional analyses such as metal speciation, bioavailable fractions, or ecological risk indices.

### 4.3 Influence of biogas slurry on crop yield and the correlation between yield and soil nutrients

The higher soybean yield observed under the combined biogas slurry and mineral fertilizer treatment (Figure 3) suggests a synergistic effect between organic and inorganic nutrient sources. This synergy arises from temporal complementarity: mineral fertilizers deliver immediate nutrient availability during early vegetative stages, while biogas slurry sustains nutrient release throughout pod formation and seed filling (Lin et al., 2022). The gradual mineralization of organic N and P in the biogas slurry ensures a continuous nutrient supply, aligning better with the soybean’s physiological demand curve. This nutrient synchronization enhances leaf chlorophyll content, nitrogen assimilation, and photosynthetic activity, leading to improved reproductive success and grain yield (Engedal et al., 2025). In addition, the presence of organic matter and enhanced microbial activity under the combined regime may improve soil structure and root development, thereby enhancing access to water and nutrients in the rhizosphere (Zhang et al., 2021). Such improvements in rooting depth and fine-root proliferation are particularly important in pot systems and under fluctuating moisture, where nutrient diffusion and mass flow can constrain uptake. In contrast, the biogas slurry-only treatment produced lower yield gains compared with the control. This outcome may be associated with the slower mineralization rate of organic nitrogen, which could delay nitrate availability during early critical growth phases (Shi et al., 2026). During these stages, insufficient mineral N may limit chlorophyll synthesis, reduce canopy expansion, and weaken root development, leading to fewer pods per plant and lower final yield (Kumar et al., 2023; Qiang et al., 2025). Once mineralization accelerates later in the season, much of the nitrogen may be released when the plant’s nutrient demand declines, potentially reducing nitrogen-use efficiency. Moreover, if early N supply is suboptimal, nodulation and biological N fixation may not fully compensate for the initial deficit, which could contribute to reduced yield performance (Sun et al., 2025). These limitations can be mitigated through strategies such as split applications, co-composting, or integration with nitrification inhibitors to better synchronize N release with plant uptake dynamics. Optimizing biogas slurry timing relative to key phenological stages (e.g., V3–V5 and R1–R3) could further enhance its contribution to soybean productivity. This result is consistent with previous studies showing that combined organic-mineral fertilization often outperforms sole organic fertilization, as it better synchronizes nutrient release with crop demand (Liu et al., 2026). In contrast to field-based studies, where residual effects of organic amendments may accumulate over time, the lower performance of biogas slurry alone in this short-term pot experiment may reflect the limited time available for nutrient mineralization and plant uptake. In addition, these interpretations are based on indirect evidence and should be confirmed by direct measurements of soil mineral nitrogen and plant nitrogen uptake in future studies.

The observed positive correlations between grain yield, leaf N content, chlorophyll, and MBP in our study highlight the tight coupling between nutrient status and soybean productivity. Higher leaf N and chlorophyll values were consistently associated with increased yield, indicating that improved N nutrition and photosynthetic capacity directly support biomass accumulation and pod and seed formation (Bassi et al., 2018; Wang G. et al., 2022). Likewise, the strong positive relationship between yield and MBP suggests that soils with larger microbial P pools are better able to sustain plant-available P during critical reproductive stages, resulting in higher grain production (García-berumen et al., 2025). Together, these correlations show that both plant N status (as reflected in leaf N and chlorophyll) and soil P dynamics (as reflected in MBP) are key determinants of yield across the different fertilization regimes. This pattern confirms that fertilization strategies that enhance nutrient availability and retention in both soil and plant compartments are most effective in maximizing soybean yield in black soils. Similar relationships between plant N status, microbial P pools, and yield have been reported in recent crop nutrition studies, supporting the view that productive response depends not only on total nutrient input but also on the efficiency of nutrient transformation and plant acquisition (Pradhan et al., 2026).

These results further suggest that, beyond direct nutrient supply, microbial-mediated processes may contribute to nutrient availability and plant performance, highlighting the importance of examining microbial community dynamics under different fertilization regimes.

### 4.4 Impact of fertilizer application on soil microbial community structure and diversity

Microbial community structure was further analyzed to provide mechanistic insight into the observed changes in soil properties and crop yield under different fertilization treatments. The structure and diversity of soil microbial communities were profoundly influenced by the type of fertilizer applied, reflecting the selective pressures imposed by different nutrient environments. Biogas slurry and the mixed fertilizer enriched oligotrophic bacterial phyla such as *Acidobacteriota, Gemmatimonadota*, and *Chloroflexi*, taxa commonly associated with efficient decomposition of complex organic substrates (Li D. et al., 2022). These groups are functionally important for sustaining carbon cycling under nutrient-limited conditions, as they produce extracellular enzymes that depolymerize lignin-derived carbon and aromatic compounds. Their enrichment suggests that biogas slurry promotes a microbial community structure potentially associated with enhanced carbon turnover through microbial residue formation and humus synthesis (Gonçalves et al., 2024; Kalam et al., 2020). In addition, the higher relative abundance of these phyla under biogas slurry-based treatments indicates a shift away from short-term, fast-growing copiotrophs toward communities better adapted to gradual nutrient release and complex carbon turnover (Feng et al., 2024). This shift is consistent with the observed increases in SOC and MBC and may help explain the improved soil nutrient dynamics and plant performance observed under biogas slurry-based treatments. These patterns are further supported by the PCoA analysis, which showed only partial separation among bacterial communities across treatments, indicating that bacterial community structure was moderately influenced by fertilization practices. Recent microbiome studies have likewise reported enrichment of *Acidobacteriota*, *Chloroflexi*, and other oligotrophic taxa under organic amendment regimes, explaining complex supply of carbon substrates (Walia et al., 2026). Compared with mineral-fertilized soils, this shift suggests that biogas slurry promotes a microbial assemblage more closely associated with gradual decomposition and carbon conservation (Yin et al., 2025). In contrast, the decline of *Proteobacteria* and *Bacteroidota*, typically dominant under nutrient-rich mineral fertilization, indicates a shift from copiotrophic to oligotrophic dominance. This transition reflects a move toward more energy-efficient microbial communities that rely on slow but steady decomposition processes rather than rapid mineral nutrient turnover (Feng et al., 2024; Finn et al., 2020). Such microbial structures enhance soil organic matter stabilization and resilience against environmental fluctuations, characteristics that may contribute to improved soil functioning (Hu et al., 2025). This trend is consistent with recent reports that nutrient-rich mineral fertilization tends to favor fast-growing copiotrophic taxa. In contrast, organic inputs support communities adapted to slower but more stable substrate turnover (Yu et al., 2026).

The fungal community exhibited similar fertilizer-dependent restructuring, with biogas slurry promoting *Ascomycota* and *Fungi_phy_incertae_sedis*, taxa associated with lignocellulosic organic matter decomposition (Su et al., 2025). These groups contribute to the breakdown and transformation of complex organic substrates, supporting carbon stabilization processes in soil (Li Y. et al., 2025). The enrichment of these taxa suggests that biogas slurry may promote fungal-mediated nutrient cycling processes and organic matter transformation (Cai et al., 2024). Similar enrichment of lignocellulose-degrading fungal groups under organic amendments has been observed in recent studies, where fungal restructuring was associated with improved residue decomposition and organic matter transformation (Ma et al., 2026). In the present study, this response further suggests that biogas slurry may support fungal-mediated processes important for maintaining black soil quality. This observation is consistent with the PCoA results, where fungal communities exhibited a clearer separation among treatments, particularly under biogas slurry application, highlighting their higher sensitivity to fertilization compared with bacterial communities.

Taken together, these microbial responses indicate that biogas slurry not only altered nutrient pools but also shifted microbial community composition toward taxa associated with complex organic matter decomposition and more balanced nutrient turnover. Similar community restructuring has been reported in recent studies of organic amendment application, although the magnitude of the response depends on soil type, amendment characteristics, and experimental duration (Forsmark et al., 2024). In the present study, these shifts suggest that biogas slurry can support microbial processes linked to soil functioning under the tested conditions. The Venn diagram analysis further supports these observations by showing a higher number of unique OTUs under biogas slurry treatment compared with other fertilization regimes. This pattern suggests that biogas slurry may promote the enrichment of specific microbial taxa, thereby increasing community differentiation (Walia et al., 2026). In contrast, the relatively lower number of unique OTUs under mixed fertilization indicates a more selective environment, where combined nutrient inputs may favor a narrower range of microbial groups. The heatmap analysis further confirmed these trends by showing distinct clustering of fungal communities under biogas slurry and combined treatments, whereas bacterial communities displayed more moderate differentiation. Together, these results indicate that fertilization, particularly biogas slurry application, induces stronger compositional and structural shifts in fungal communities than in bacterial communities.

### 4.5 Study limitations

Although this study provides valuable insights into the short-term effects of biogas slurry on black soil properties and soybean productivity, several limitations should be acknowledged.

The experiment was conducted under controlled pot conditions, which may not fully represent field conditions like soil heterogeneity, root distribution, rainfall variability, and complex biotic interactions influencing nutrient cycling and microbial processes. Pot experiments may restrict root development and alter soil moisture dynamics, potentially affecting plant-soil interactions compared with field conditions.

Moreover, the experiment duration was relatively short and primarily reflects immediate responses of soil chemical and biological properties to treatments. Long-term processes such as soil organic carbon accumulation, microbial community stabilization, and trace metal dynamics typically require multi-season or multi-year observations for accurate evaluation. Therefore, caution should be exercised when extrapolating these results beyond the experimental conditions.

In addition, the experiment included a limited number of replications, which may reduce the sensitivity for detecting subtle differences among treatments and limit the ability to capture broader environmental variability. This experimental design reflects standard practices in controlled pot studies, but broader-scale field experiments in future research are needed. Noticeably, microbial community composition was characterized, functional processes were inferred from taxonomic profiles rather than directly assessed through enzyme activities or functional gene expressions. However, comprehensive ecological analyses integrating taxonomic composition with environmental variables could further improve the understanding of community-environment interactions. This aspect could represent an important direction for future research.

Finally, the evaluation of heavy metal dynamics was limited to short-term changes in total soil concentrations and did not include analyses of metal bioavailability, speciation, or plant tissue accumulation. Mineral soil nitrogen dynamics during the experimental period were not directly measured, and therefore, interpretations related to nitrogen availability and mineralization should be considered as indicative rather than conclusive. Future studies should incorporate bioavailable heavy metal fractions, plant uptake measurements, and ecological risk indices (e.g., geoaccumulation index or pollution load index) to better assess the environmental implications of biogas slurry application. Overall, long-term field experiments are necessary to determine the patterns under diverse agricultural conditions to evaluate potential environmental risks.

## 5 Conclusion

This study evaluated the short-term effects of different fertilization strategies on soil chemical properties, microbial indicators, and soybean productivity in black soils of Northeast China under controlled pot conditions. The results indicate that biogas slurry and mixed fertilizer treatments increased soil organic carbon and total nitrogen compared with chemical fertilizer alone. In addition, biogas slurry partially mitigated soil acidification observed under mineral fertilization and enhanced microbial biomass indicators. Although biogas slurry alone improved soil health indicators, it resulted in lower soybean yield compared with mixed fertilization. In contrast, the combined application of biogas slurry and mineral fertilizer was associated with higher crop productivity and improved soil nutrient status, suggesting a potential complementary effect between organic and inorganic nutrient sources. Biogas slurry application also increased soil Cu and Cd concentrations, highlighting the need for careful management. Furthermore, fertilization treatments influenced microbial community structure, indicating potential links between soil biological processes and nutrient dynamics under the conditions tested. Overall, these findings suggest that integrating biogas slurry and inorganic fertilizers may contribute to improved soil management in black soil systems. However, the results should be interpreted within the context of this short-term pot experiment. Long-term field studies are required to confirm the persistence of these effects and to assess potential environmental risks associated with biogas slurry application.

## Data Availability

The sequencing data generated in this study have been deposited in the NCBI Sequence Read Archive (SRA) under BioProject accession number PRJNA1453179 (https://www.ncbi.nlm.nih.gov/bioproject/PRJNA1453179).
